# Distinct Contributions of Genes and Environment to Visual Size Illusion and the Underlying Neural Mechanism

**DOI:** 10.1093/cercor/bhab262

**Published:** 2021-08-11

**Authors:** Lihong Chen, Qian Xu, Li Shen, Tian Yuan, Ying Wang, Wen Zhou, Yi Jiang

**Affiliations:** Research Center of Brain and Cognitive Neuroscience, Liaoning Normal University, Dalian 116029, P.R. China; Key Laboratory of Brain and Cognitive Neuroscience, Liaoning Province, Dalian 116029, P.R. China; State Key Laboratory of Brain and Cognitive Science, CAS Center for Excellence in Brain Science and Intelligence Technology, Institute of Psychology, Chinese Academy of Sciences, Beijing 100101, P.R. China; State Key Laboratory of Brain and Cognitive Science, CAS Center for Excellence in Brain Science and Intelligence Technology, Institute of Psychology, Chinese Academy of Sciences, Beijing 100101, P.R. China; Department of Psychology, University of Chinese Academy of Sciences, Beijing 100049, P.R. China; Chinese Institute for Brain Research, Beijing 102206, P.R. China; State Key Laboratory of Brain and Cognitive Science, CAS Center for Excellence in Brain Science and Intelligence Technology, Institute of Psychology, Chinese Academy of Sciences, Beijing 100101, P.R. China; Department of Psychology, University of Chinese Academy of Sciences, Beijing 100049, P.R. China; Chinese Institute for Brain Research, Beijing 102206, P.R. China; State Key Laboratory of Brain and Cognitive Science, CAS Center for Excellence in Brain Science and Intelligence Technology, Institute of Psychology, Chinese Academy of Sciences, Beijing 100101, P.R. China; Department of Psychology, University of Chinese Academy of Sciences, Beijing 100049, P.R. China; Chinese Institute for Brain Research, Beijing 102206, P.R. China; State Key Laboratory of Brain and Cognitive Science, CAS Center for Excellence in Brain Science and Intelligence Technology, Institute of Psychology, Chinese Academy of Sciences, Beijing 100101, P.R. China; Department of Psychology, University of Chinese Academy of Sciences, Beijing 100049, P.R. China; Chinese Institute for Brain Research, Beijing 102206, P.R. China; State Key Laboratory of Brain and Cognitive Science, CAS Center for Excellence in Brain Science and Intelligence Technology, Institute of Psychology, Chinese Academy of Sciences, Beijing 100101, P.R. China; Department of Psychology, University of Chinese Academy of Sciences, Beijing 100049, P.R. China; Chinese Institute for Brain Research, Beijing 102206, P.R. China; State Key Laboratory of Brain and Cognitive Science, CAS Center for Excellence in Brain Science and Intelligence Technology, Institute of Psychology, Chinese Academy of Sciences, Beijing 100101, P.R. China; Department of Psychology, University of Chinese Academy of Sciences, Beijing 100049, P.R. China; Chinese Institute for Brain Research, Beijing 102206, P.R. China; Institute of Artificial Intelligence, Hefei Comprehensive National Science Center, Hefei 230088, P.R. China

**Keywords:** behavioral genetics, Ebbinghaus illusion, fNIRS, functional connectivity, twin study

## Abstract

As exemplified by the Ebbinghaus illusion, the perceived size of an object can be significantly biased by its surrounding context. The phenomenon is experienced by humans as well as other species, hence likely evolutionarily adaptive. Here, we examined the heritability of the Ebbinghaus illusion using a combination of the classic twin method and multichannel functional near-infrared spectroscopy. Results show that genes account for over 50% of the variance in the strength of the experienced illusion. Interestingly, activations evoked by the Ebbinghaus stimuli in the early visual cortex are explained by genetic factors whereas those in the posterior temporal cortex are explained by environmental factors. In parallel, the feedforward functional connectivity between the occipital cortex and the temporal cortex is modulated by genetic effects whereas the feedback functional connectivity is entirely shaped by environment, despite both being significantly correlated with the strength of the experienced illusion. These findings demonstrate that genetic and environmental factors work in tandem to shape the context-dependent visual size illusion, and shed new light on the links among genes, environment, brain, and subjective experience.

## Introduction

The perceived size of an object is not always a faithful representation of its physical size and is often biased by the spatial contexts surrounding that object. Such contextual modulation can be easily demonstrated by visual size illusions. For instance, in the Ebbinghaus illusion, an object would be perceived as larger when surrounded by small items than when the identical object is surrounded by large items. Converging evidence reveals that size illusions can be observed among many other species, including bottlenose dolphins (Murayama et al. 2012), redtail splitfins (Sovrano et al. 2015, 2016), rhesus macaques (Tudusciuc and Nieder 2010), gray parrots (Pepperberg et al. 2008), and even 4-day-old domestic chicks (Rosa-Salva et al. 2013). These animals experience the size illusions in analogous ways as humans do, suggesting the existence of conserved mechanisms in different taxonomic groups of animals (Vallortigara 2004, 2006; Rosa-Salva et al. 2014). In other words, the context-dependent visual size illusion might be acquired by means of evolution.

In line of this view, empirical evidence from human studies suggests that the mechanisms underlying certain size illusions might not depend entirely on visual experience, despite that postnatal environment undoubtedly plays a major role in shaping our visual processing (Zhou et al. 2010; Bao et al. 2018). For example, congenitally blind children demonstrate susceptibility to the Ponzo and Müller-Lyer illusions immediately following cataract surgery in just one eye (Gandhi et al. 2015). Similarly, congenitally blind adults exhibit the haptic Müller-Lyer illusion (Heller et al. 2002), to an extent comparable to the visual illusion in the seeing controls (Tsai 1967). Moreover, Coren and Porac (1979) observed significant correlations of the Ebbinghaus illusion strength along parent and offspring, but not along siblings. These results suggest that the neural substrates involved in visual perception of size illusions could be, at least partially, experience-independent. Perhaps evolutionary pressures lead to the innate structures of the nervous system in a way best suited for a species to perceive its environment in an adaptive way (Geisler and Kersten 2002).

To examine to what extent the context-dependent visual size illusion and the underlying neural mechanism are accounted for by genetic and environmental influences, here we conducted a twin study using the Ebbinghaus illusion and multichannel functional near-infrared spectroscopy (fNIRS). The fNIRS technique provides balanced temporal and spatial resolutions for the current study and offers an affordable measurement for a relatively large sample of participants (*N* = 160). With the twin design, we were able to employ individual differences to estimate the genetic and environmental influences on the observed phenotypes, based on the principle that monozygotic (MZ) twins and dizygotic (DZ) twins share the environmental influence to the same degree, whereas MZ twins (who share 100% of their genes) share more genes than DZ twins (who share 50% on average) and thus should be more similar in heritable traits (Wang et al. 2018). We expected that genetic and environmental influences on the visual processing of the Ebbinghaus illusion would manifest in observers’ perceived illusory strength as well as the neural computations across visual cortical areas that are critically involved in context-dependent visual size illusion. Previous studies have demonstrated that visual size information is encoded and computed to a large extent along the ventral visual stream, including V1 (Murray et al. 2006; Fang et al. 2008; Schwarzkopf et al. 2011; Sperandio et al. 2012; Pooresmaeili et al. 2013), the extrastriate cortex (Frassinetti et al. 1999; Kreutzer et al. 2015), the lateral occipital complex (Weidner and Fink 2007; Mancini et al. 2011), and the temporal cortex (Hart et al. 1992).

## Materials and Methods

### Participants

A total of 80 pairs of same-gender twins (80 male and 80 female) with a mean age of 19.74 years (between 15 and 25 years), consisting of 40 pairs of MZ twins (40 male and 40 female) and 40 pairs of DZ twins (40 male and 40 female), were recruited for payment from a twin database (Beijing Twin Study) maintained by the Institute of Psychology, Chinese Academy of Sciences (IPCAS). Sample size was determined by the G*Power statistical software (Faul et al. 2007) to be sufficient to detect a medium-sized effect (*d* ≥ 0.8), at a power larger than 95%. There were no significant difference regarding the distributions of gender and age (20.10$\pm$2.67 vs. 19.38$\pm$2.19, *t*[78] = 1.33, *P* = 0.188) between DZ and MZ twin groups. Zygosity was determined by DNA geotyping on 9 short-tandem-repeat loci, with near-100% classification accuracy. All had normal or corrected-to-normal vision and gave written, informed consent in accordance with procedures and protocols approved by the institutional review board of the IPCAS, and the study adhered to the tenets of the Declaration of Helsinki. All participants were naive to the purpose of the experiment.

### Apparatus, Stimuli, and Procedure

**Figure 1:**
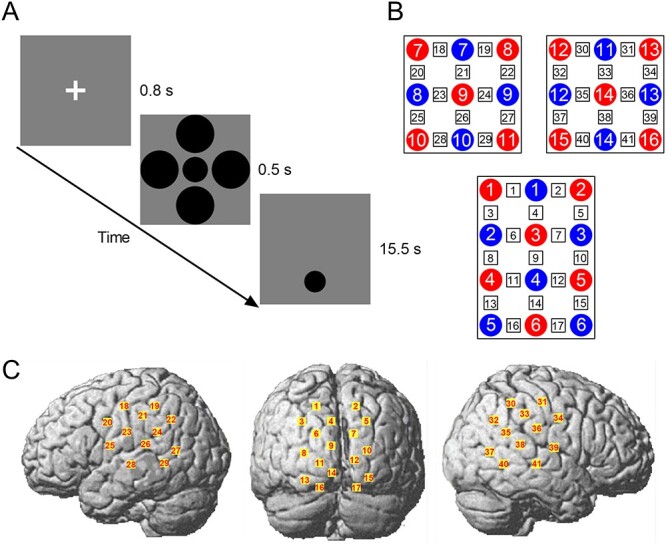
Experimental procedure and channel arrangement. (*A*) A schematic diagram of the experiment, (*B*) the arrangement of emitter (red) and detector (blue) probes providing 41 channels (black), and (*C*) the corresponding channels on the brain surface.

Stimuli were generated using Matlab (Mathworks) together with the Psychophysics Toolbox (Brainard 1997; Pelli 1997). Participants viewed an LCD monitor (1440 × 900, 60 Hz) binocularly from a distance of 57 cm. A chin rest was used to stabilize head position. A target circle (1.14${}^{\circ}\times$1.14${}^{\circ}$) surrounded by 4 large (1.71${}^{\circ}\times$1.71${}^{\circ}$) or small (0.57${}^{\circ}\times$0.57${}^{\circ}$) circles was presented for 0.5 s, followed by a comparison circle presented below the illusory configuration with a period of 15.5 s (4.28° from the monitor center; see Fig. 1*A*). The initial size of the comparison circle (0.91°–$1.37{}^{\circ}$) varied from trial to trial in steps of 0.06${}^{\circ}$. Participants were asked to adjust the size of the comparison circle to match that of the target. The target and the comparison circle had neither temporal nor spatial overlap. There were a total of 36 trials with 18 repetitions for each condition. In order to minimize any potential confounding influences, each pair of twins (either MZ or DZ) came together to the lab and completed the task.

### fNIRS Data Acquisition

Relative changes in oxygenated hemoglobin (oxy-Hb), deoxygenated hemoglobin and total hemoglobin were measured using 780, 805, and 830-nm wavelengths of near-infrared light on the basis of the modified Beer–Lambert law (arbitrary units, mM$\bullet$mm). Measurements were performed on a continuous wave system (LABNIRS, Shimadzu Co.) using two 3$\times$3 and one 4$\times$3 optode probe sets (consisting of 16 emitter probes and 14 detector probes) that provided a total of 41 channels separately by 3.0 cm (Fig. 1*B*) and allowed for the measurement of neural activity ~15-mm beneath the scalp (Fukui et al. 2003). The arrangement of the probes covered bilateral temporal and occipital cortical surface regions. The lowest probes in the occipital region were located along the O1–O2 line according to the international 10–20 system in EEG. Resistance was measured for each channel before recording to ensure acceptable signal-to-noise ratios, and adjustments were made until all channels met the minimum criteria established by the LABNIRS recording standards.

The data sampling rate was 47.62 Hz. We recorded 3-min brain activities in resting state before the experiment during which participants were required to close their eyes, as well as task-related brain activities when the participants performed the size matching task.

### Optode Localization

The anatomical locations of channels in relation to standard head landmarks, including nasion, top center, left tragus, and right tragus, were determined for two participants using a 3D Digitizer (Fastrak; Polhemus). The Montreal Neurological Institute (MNI) coordinates (Mazziotta et al. 2001) for the channels were obtained by using the NIRS-SPM software (Ye et al. 2009) with Matlab, and the corresponding anatomical locations of each channel were determined by the provided atlas (Rorden and Brett 2000). The locations of channels (Fig. 1*C*) were probabilistically estimated and anatomically labeled in the standard brain space (LONI Probabilistic Brain Atlas 40, LBPA40) according to Tsuzuki et al. (2007).

### fNIRS Data Processing

Concentration change in oxy-Hb is a more sensitive and reliable measure than deoxygenated or total hemoglobin concentration change and has been widely used in previous fNIRS studies (Strangman et al. 2002; Sakakibara et al. 2014; Hyde et al. 2018). Therefore, we focused on concentrations of oxy-Hb in the data analyses. For each participant, raw data were band-pass filtered (0.01–0.5 Hz) to attenuate potential noise confusion including respiration and cardiac cycle effects (Heinzel et al. 2013; Brigadoi et al. 2014). A principal component analysis was used to remove motion artifacts (Zhang et al. 2016; Hirsch et al. 2017). In the following, the data were normalized by subtracting the mean signal 0.5 s before the onset of illusory configuration for each channel and for each condition. Any channel without a signal due to insufficient optode contact with the scalp was identified automatically by the root mean square of the raw data when the magnitude was more than 10 times greater than the average signal (Hirsch et al. 2017). Approximately 9.92% of the channels in the entire data set were automatically removed prior to subsequent analyses based on this criterion. For the resting-state data, the data 20 s both at the beginning and at the end of the run were excluded to obtain stable signals. Similar methods were used to reduce potential noise and motion artifacts (15.44% data were excluded from further analysis). Two main regions of interest (ROIs), that is, the early visual cortex and the left posterior temporal cortex, were identified based on the task-related data where the peak oxy-Hb within 16 s after the onset of illusory configuration was measured. We also performed time-course correlation between these two ROIs with time lags of $\pm$1 s, and then transformed the *r* values to *z* values by 0.5$\times{\mathit{\log}}_{10}^{[(1+r)/(1-r)]}$ for further analysis.

**Figure 2:**
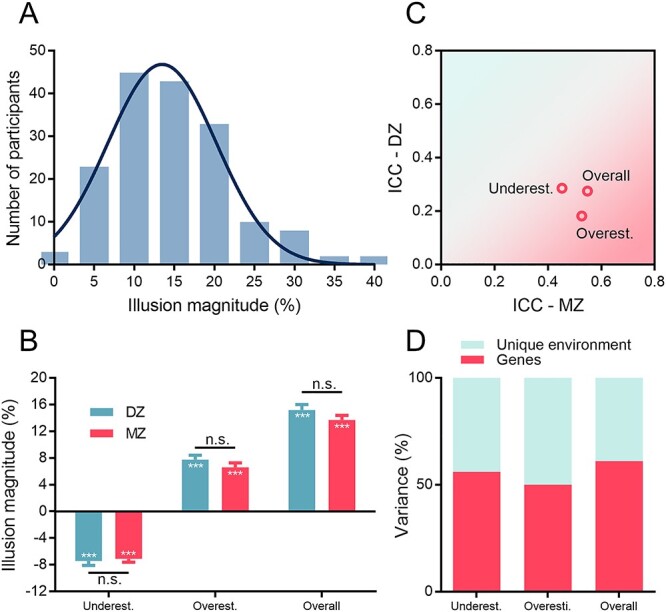
Behavioral results. (*A*) Distribution of the overall illusion magnitude for a group of 160 participants, (*B*) mean illusion magnitude for DZ and MZ twins, (*C*) mean intraclass correlation coefficients (ICCs) for the members of DZ and MZ twin pairs, and (*D*) genetic and environmental contributions to the variance in the illusion effects. Error bars represent one standard error of the mean.

### Genetic Modeling Analysis

Using intraclass correlation analysis, we measured the resemblance between the members within MZ and DZ twin pairs, respectively. By comparing the MZ and DZ correlations in a twin design, we could estimate the relative influences of genes and environment on individual variation in a specific phenotype. Structural equation modeling was applied to estimate the contributions of additive genetic (A), common (C), and unique (E) environmental factors. After fitting the full ACE model to the data, we also separately tested the AE, CE, and E submodels. Chi-square statistics were used to examine the goodness of fit for each model and to compare the submodels with the saturated models to assess the contribution of the dropped factors. Subsequently, we estimated the heritability of a phenotype using the best model selected based on both the goodness of fit and parameter parsimony according to the Akaike information criterion (AIC; Wang et al. 2020). The modeling analysis was performed using the statistical package Mx (http://www.vcu.edu/mx/).

## Results

### Genetic Contribution to Perceived Visual Size Illusion

The overall visual illusory strength was calculated as the perceived size disparity of the same target surrounded by small (i.e., the overestimation portion) and large (i.e., the underestimation portion) inducers. Trials with extreme values outside $\pm$2.5 SD (1.68% of all trials) were excluded from further analyses. The perceived illusory strength varied across participants, illustrating individual variability in contextual modulation of visual size perception (Fig. 2*A*). Both DZ and MZ twins showed significant underestimation (DZ: *t*[79] = −11.29, *P* < 0.001, *d* = 1.26; MZ: *t*[79] = −13.96, *P* < 0.001, *d* = 1.56) and overestimation effects (DZ: *t*[79] = 11.32, *P* < 0.001, *d* = 1.27; MZ: *t*[79] = 9.84, *P* < 0.001, *d* = 1.10), as well as the overall illusion effect (DZ: *t*[79] = 17.85, *P* < 0.001, *d* = 1.20; MZ: *t*[79] = 20.09, *P* < 0.001, *d* = 2.25). Moreover, there were no significant differences between these two groups in terms of the illusion magnitudes (the underestimation portion: −7.44% vs. −7.11%, *t*[79] = −0.38, *P* > 0.250, *d* = 0.04; the overestimation portion: 7.74% vs. 6.59%, *t*[79] = 1.42, *P* = 0.159, *d* = 0.16; and the overall illusion effect: 15.17% vs. 13.70%, *t*[79] = 1.40, *P* = 0.166, *d* = 0.16; see Fig. 2*B*).

The magnitudes of the underestimation portion, the overestimation portion, and the overall illusion effect were then used as phenotypic variables. Intraclass correlation analysis revealed that the similarity of members within MZ twin pairs was larger than that within DZ twin pairs in the underestimation (MZ: *r* = 0.45, 95% confidence interval [CI] = [0.17, 0.67], *P* = 0.001; DZ: *r* = 0.29, 95% CI = [0.02, 0.55], *P* = 0.034) and the overestimation (MZ: *r* = 0.53, 95% CI = [0.26, 0.72], *P* < 0.001; DZ: *r* = 0.18, 95% CI = [−0.13, 0.46], *P* = 0.126) portions, as well as the overall illusion effect (MZ: *r* = 0.55, 95% CI = [0.29, 0.73], *P* < 0.001; DZ: *r* = 0.28, 95% CI = [−0.03, 0.54], *P* = 0.040; see Fig. 2*C*). To quantify the respective contributions of genes and environment, we submitted the data to the ACE genetic modeling analysis (Table 1). The heritability, that is, the proportion of variance that can be accounted for by genetic factors, was estimated to be 56% (95% CI = [30%, 73%]), 50% (95% CI = [25%, 69%]), and 61% (95% CI = [36%, 77%]) for the underestimation portion, the overestimation portion, and the overall illusion effect, respectively (Fig. 2*D*).

**Table 1:** The goodness-of-fit statistics for the full and best-fitting models with phenotypes of the behavioral illusion effects, task-related brain activities in the occipital and the temporal cortices, and the functional connectivity between these two brain regions

	Model		*a* ^2^ (95% CI)	*c* ^2^ (95% CI)	*e* ^2^ (95% CI)	AIC
Behavioral effect	Full					
Underestimation		ACE	0.54 (0–0.73)	0.01 (0–0.49)	0.45 (0.27–0.75)	−1.64
Overestimation		ACE	0.50 (0–0.69)	0 (0–0.42)	0.50 (0.31–0.75)	−4.11
Total illusion		ACE	0.61 (0.10–0.77)	0 (0–0.36)	0.39 (0.23–0.64)	0.50
Neural underestimation						
Channel #4		ACE	0 (0–0.38)	0.25 (0–0.47)	0.75 (0.53–0.99)	2.05
Channel #9		ACE	0 (0–0.40)	0.12 (0–0.39)	0.88 (0.60–1)	−2.40
Channel #23		ACE	0 (0–0.33)	0.06 (0–0.31)	0.94 (0.67–1)	−4.72
Channel #26		ACE	0 (0–0.47)	0.29 (0–0.51)	0.71 (0.49–0.98)	−3.89
Neural overestimation						
Channel #4		ACE	0.36 (0–0.61)	0.03 (0–0.49)	0.61 (0.39–0.91)	−1.22
Channel #9		ACE	0.35 (0–0.67)	0 (0–0.31)	0.65 (0.33–1)	−1.07
Channel #23		ACE	0.23 (0–0.56)	0 (0–0.36)	0.77 (0.44–1)	5.16
Channel #26		ACE	0.07 (0–0.61)	0.28 (0–0.55)	0.65 (0.39–0.92)	−4.05
Functional connectivity						
Plus 1-s lag		ACE	0.34 (0–0.61)	0.02 (0–0.48)	0.64 (0.39–0.97)	0.04
Minus 1-s lag		ACE	0 (0–0.45)	0.14 (0–0.39)	0.86 (0.55–1)	−4.28
Behavioral effect	Best					
Underestimation		AE	0.56 (0.30–0.73)	—	0.44 (0.27–0.70)	−3.64
Overestimation		AE	0.50 (0.25–0.69)	—	0.50 (0.31–0.75)	−6.11
Total illusion		AE	0.61 (0.36–0.77)	—	0.39 (0.23–0.64)	−1.51
Neural underestimation						
Channel #4		CE	—	0.25 (0.01–0.47)	0.75 (0.53–0.99)	0.05
Channel #9		E	—	—	1 (1–1)	−5.65
Channel #23		E	—	—	1 (1–1)	−8.54
Channel #26		CE	—	0.29 (0.02–0.51)	0.71 (0.49–0.98)	−5.89
Neural overestimation						
Channel #4		AE	0.39 (0.10–0.61)	—	0.61 (0.39–0.90)	−3.21
Channel #9		AE	0.35 (0–0.67)	—	0.65 (0.33–1)	−3.07
Channel #23		E	—	—	1 (1–1)	2.47
Channel #26		CE	—	0.34 (0.08–0.55)	0.66 (0.45–0.92)	−6.03
Functional connectivity						
Plus 1-s lag		AE	0.36 (0.04–0.61)	—	0.64 (0.39–0.96)	−1.96
Minus 1-s lag		E	—	—	1 (1–1)	−7.15

### Genes and Environment Mutually Contribute to Task-Related Oxygenated Hemoglobin (Oxy-Hb) Responses

According to previous studies (Murray et al. 2006; Fang et al. 2008; Pooresmaeili et al. 2013; Weidner et al. 2014), the neural processing critically involved in the context-dependent visual size perception would be expected to elicit significantly stronger oxy-Hb responses to the target with large perceived size (i.e., surrounded by small inducers) compared with the identical target with small perceived size (i.e., surrounded by large inducers). Such selection criterion would exclude most of, if not all, the brain regions that are only sensitive to physical visual size rather than visual size illusion (see Supplementary Material for the control experiment). As expected, brain regions that exhibited sensitivity to visual size illusion were primarily found in V1 (channel #9: *t*[101] = 4.41, *P* < 0.001, *d* = 0.44; Table 2), V2/V3 (channel #4: *t*[131] = 2.14, *P* = 0.034, *d* = 0.19), and the posterior temporal cortex (channel #23: *t*[115] = 2.72, *P* = 0.007, *d* = 0.25; channel #26: *t*[107] = 2.60, *P* = 0.011, *d* = 0.25). These results were further replicated by a control experiment in which 5 types of stimuli (a target surrounded by 4 large or small inducers, 4 large or small inducers only, and a target only) were tested (see Supplementary Fig. 1). Consistently, both the early visual cortex and the posterior temporal cortex exhibited significantly stronger oxy-Hb responses to the target with large perceived size compared with the identical target with small perceived size when the oxy-Hb responses to the surrounding inducers only were respectively subtracted from those to the Ebbinghaus illusion configurations (see Supplementary Material for more details), confirming the sensitivity of these brain regions to the visual size illusion effect per se rather than to the physical difference of the surrounding inducers.

**Table 2:** Cortical regions (LBPA40) and MNI coordinates of channels #4, #9, #23, and #26

Channel	Mean MNI coordinates			Anatomical region	BA	Probability
Number	*x*	*y*	*z*			
4	−5	−92	36	Visual association cortex (V2)	18	0.38
				V3	19	0.62
9	−5	−105	16	Primary visual cortex (V1)	17	0.98
				Visual association cortex (V2)	18	0.02
23	−68	−15	29	Primary somatosensory cortex	1	0.08
				Primary Somatosensory cortex	2	0.36
				Superior temporal gyrus	22	0.01
				Subcentral area	43	0.44
				Retrosubicular area	48	0.11
26	−69	−33	17	Primary somatosensory cortex	2	0.09
				Superior temporal gyrus	22	0.72
				Primary and auditory association cortex	42	0.06
				Retrosubicular area	48	0.12

**Figure 3:**
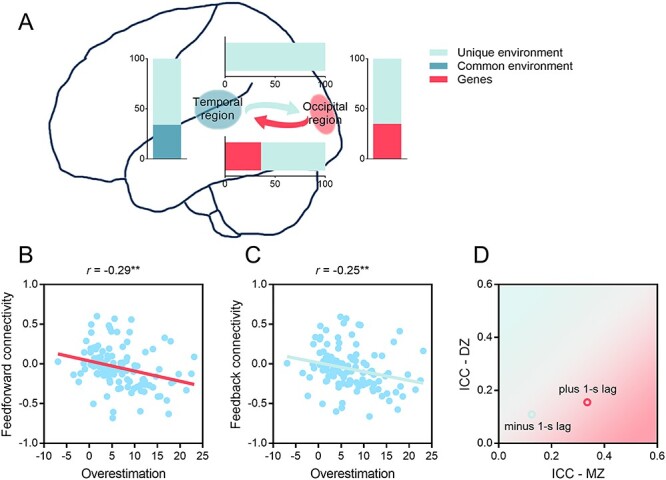
Results of oxy-Hb signals. (*A*) Genetic and environmental contributions to the variance of brain activities (the overestimation portion) in the occipital and the temporal regions and to the variance of the time-course correlations between the two ROIs for the two time-lag conditions, correlation of *z*-transformed *r* values for plus 1-s lag (*B*) and minus 1-s lag (*C*) with the overestimation portion, and (*D*) mean ICCs for DZ and MZ twin pairs as a function of time lag.

Intraclass correlation analysis revealed that, only when the target was surrounded by small inducers (the overestimation portion), similarity of oxy-Hb responses in the early visual cortex was larger within MZ twin pairs than within DZ twin pairs (channel #4: MZ: *r* = 0.37, 95% CI = [0.05, 0.62], *P* = 0.012; DZ: *r* = 0.15, 95% CI = [−0.21, 0.48], *P* = 0.204; channel #9: MZ: *r* = 0.48, 95% CI = [0.09, 0.74], *P* = 0.009; DZ: *r* = −0.11, 95% CI = [−0.45, 0.27], *P* > 0.250), but this trend was largely weakened in the posterior temporal cortex (channel # 23: MZ: *r* = 0.23, 95% CI = [−0.16, 0.55], *P* = 0.122; DZ: *r* = 0.03, 95% CI = [−0.32, 0.37], *P* > 0.250; channel #26: MZ: *r* = 0.37, 95% CI = [0.01, 0.65], *P* = 0.022; DZ: *r* = 0.33, 95% CI = [−0.06, 0.63], *P* = 0.045). To quantify the respective contributions of genes and environment, we submitted the data into the ACE genetic model. When the target was surrounded by small inducers, the heritability of the overestimation portion in the early visual cortex was estimated to be 39% (channel #4, 95% CI = [10%, 61%]) and 35% (channel #9, 95% CI = [0%, 67%]; see Fig. 3*A*), respectively. In the posterior temporal cortex (channel #26), common environmental factors could account for 34% (95% CI = [8%, 55%]; see Fig. 3*A*) of the overall variance of oxy-Hb responses to the overestimation portion. Different from the behavioral observation, there was no evidence of genetic influences on the underestimation portion in the early visual cortex or in the posterior temporal cortex, with the percentages of the overall variance of oxy-Hb responses attributable to common environment being 25% (channel #4, 95% CI = [1%, 47%]) and 29% (channel #26, 95% CI = [2%, 51%]), respectively. This pattern of results suggests that the underestimation and the overestimation portions might be supported by different neural mechanisms. Considering that brain responses only in the visual cortex and parts of the parietal and the temporal regions were recorded (a limited number of channels were available for simultaneous recording), it is possible that the underestimation portion of the Ebbinghaus illusion engages critical neural processing in other brain regions (e.g., the frontal cortex; Kreutzer et al. 2015) than the recorded sites, which might account for the heritability observed with the behavioral underestimation effect.

### Genetic and Environmental Influences on Feedforward and Feedback Functional Connectivity between the Early Visual Cortex and the Posterior Temporal Cortex

Genetic modeling analyses have revealed that activations evoked by the overestimation portion of the Ebbinghaus illusion in the early visual cortex are accounted for by genetic effects whereas those in the posterior temporal cortex are explained by environmental factors. It remained to be explored whether these two brain regions are functionally connected to mediate the perceptual illusion effect and how this connectivity, if observed, is modulated by genetic and environmental factors. Since the oxy-Hb response patterns obtained from the proximate channels (i.e., channels #4 and #9 in the early visual cortex and channels #23 and #26 in the posterior temporal cortex) were quite similar, data from these channels were respectively combined and defined as ROIs. We calculated the time-course correlations between these two ROIs (ROI 1: channels #4 and #9; ROI 2: channels #23 and #26) by using time lags of $\pm$1 s, with plus 1-s lag representing the feedforward functional connectivity between ROI 1 (i.e., the early visual cortex) and ROI 2 (i.e., the posterior temporal cortex) and minus 1-s lag representing the feedback functional connectivity. The functional connectivity was significant between the two ROIs for both plus 1-s lag (*t*[113] = −2.02, *P* = 0.045, *d* = 0.19) and minus 1-s lag (*t*[113] = −2.49, *P* = 0.014, *d* = 0.23) conditions. Moreover, there was no significant difference between DZ and MZ groups for the two time-lag conditions (plus 1-s lag: *t*[112] = −0.67, *P* > 0.250, *d* = 0.13; minus 1-s lag: *t*[112] = −1.06, *P* > 0.250, *d* = 0.20). Notably, the *z*-transformed *r* values were negatively correlated with the behavioral overestimation portion (plus 1-s lag: *r*[114] = −0.29, *P* = 0.002; minus 1-s lag: *r*[114] = −0.25, *P* = 0.007; see Fig. 3*B* and *C*).

Intraclass correlation analysis of the *z*-transformed *r* values between ROI 1 and ROI 2 revealed that the similarity of members within MZ twin pairs (*r* = 0.34, 95% CI = [−0.03, 0.62], *P* = 0.03) was larger than that within DZ twin pairs (*r* = 0.16, 95% CI = [−0.22, 0.49], *P* = 0.208) for the plus 1-s lag (Fig. 3*D*), and genes could explain 36% (95% CI = [4%, 61%]; see Fig. 3*A*) of the observed variance, estimated by the best-fitting genetic model with goodness of fit of the AE model: χ^2^(4) = 6.05, *P* = 0.20, AIC = −1.96. However, the ICC for the minus 1-s lag was neither evident within MZ pairs (*r* = 0.13, 95% CI = [−0.24, 0.46], *P* > 0.250) nor within DZ pairs (*r* = 0.11, 95% CI = [−0.27, 0.46], *P* > 0.250), suggesting that the feedback functional connectivity was primarily accounted for by non-genetic effects (heritability = 0%, 95% CI = [0%, 0%]), estimated by the best-fitting genetic model with goodness of fit of the E model: χ^2^(5) = 2.85, *P* = 0.724, AIC = −7.15.

## Discussion

Contextual modulation of visual size perception, as well exemplified by the Ebbinghaus illusion, is a ubiquitous visual phenomenon. Intriguingly, the visual size illusion effect, which can even take place independent of conscious awareness (Chen et al. 2018), has been observed not only in humans but also among many other species (Pepperberg et al. 2008; Tudusciuc and Nieder 2010; Murayama et al. 2012; Rosa-Salva et al. 2013; Sovrano et al. 2015, 2016), exhibiting its evolutionary significance. Moreover, perception of visual illusions has been observed in newly hatched chicks (Regolin and Vallortigara 1995; Clara et al. 2006; Regolin et al. 2011), indicating the innate nature of visual size illusions. By measuring the Ebbinghaus illusion in twin participants and recording their brain activities with fNIRS, here we demonstrated that perception of the Ebbinghaus illusion is heritable, and this heritability manifests itself in both observers’ perceived illusory strength and neural activity in the early visual cortex (for the overestimation portion). In particular, genes can explain 61% variance of the perceptual illusion effect, as well as 56% and 50% variance of the underestimation and the overestimation portions, respectively. In the early visual cortex, genes account for 39% (channel #4) and 35% (channel #9) variance of oxy-Hb responses when the target was surrounded by small inducers (the overestimation portion), whereas common environment contributed to 25% (channel #4) variance of oxy-Hb responses when the target was surrounded by large inducers (the underestimation portion). In the posterior temporal cortex (channel #26), common environment could explain 29% and 34% variance of oxy-Hb responses when the target was surrounded by large and small inducers, respectively. More importantly, genes contributed to 36% variance of the feedforward functional connectivity between the early visual cortex and the posterior temporal cortex, and this connectivity strength was significantly correlated with the overestimation portion of the perceptual illusion effect. By contrast, although the strength of the feedback functional connectivity exhibited a similar correlational pattern, this connectivity was primarily modulated by non-genetic effects.

It is commonly believed that experience-driven development of sensitivity to certain visual clues or contexts plays a major role in shaping our visual size perception including visual size illusions. For example, it has been shown that the Ebbinghaus illusion is experienced less strongly in African remote cultures and is enhanced in East Asian populations (de Fockert et al. 2007; Doherty et al. 2008; Caparos et al. 2012). On the other hand, the evidence in favor of innate nature of visual size illusions has been collected. For instance, Gandhi et al. (2015) have observed the Ponzo and Müller-Lyer illusions for newly sighted children who gain sight after extended early-onset blindness, suggesting that the susceptibility to these two size illusions does not rely on an individual’s acquired experience of the visual world, but is rather based on the innate mechanisms that are experience-independent. In the current study, we used the classic twin method to address this issue and showed that the visual perception of the Ebbinghaus illusion, including the underestimation and the overestimation portions, is heritable. The percentage of the overall variance attributable to the genetic component is higher than 50%. Therefore, our study not only confirms but also quantifies the crucial role of genes in shaping the visual processing of the Ebbinghaus illusion.

Converging evidence suggests that multiple cortical areas along the ventral stream are involved in visual size perception. Neurophysiological studies reveal that lesion of the inferior temporal cortex or the extrastriate cortex of rhesus monkeys affects their abilities in size constancy (Humphrey and Weiskrantz 1969; Ungerleider et al. 1977) and size perception (Schiller and Lee 1991). Brain-damaged patients with lesion of the extrastriate cortex or the inferior middle and superior temporal lobe show erroneous size perception (Cohen et al. 1994; Frassinetti et al. 1999; Ferber and Karnath 2001). Similarly, cortical stimulation of the left posterior middle temporal gyrus of an epilepsy patient impairs her ability to access size information when questioned verbally (Hart et al. 1992). Moreover, Weidner and Fink (2007) demonstrated that the strength of the Müller-Lyer illusion is largely associated with the involvement of the lateral occipital cortex. Their subsequent studies showed that the lateral occipital and the inferior temporal regions play an essential role in the generation of the Müller-Lyer illusion (Weidner et al. 2010), and bilateral fusiform gyrus and V1 are involved in the perception of the moon illusion (Weidner et al. 2014). Furthermore, both functional and anatomical features of V1 have been found to reflect the perceived size more than the physical size of stimuli in the context of visual illusion and afterimage (Murray et al. 2006; Fang et al. 2008; Schwarzkopf et al. 2011; Sperandio et al. 2012; Pooresmaeili et al. 2013; Schwarzkopf and Rees 2013; Wang et al. 2021). The present study resonates well with previous studies and shows that both the early visual cortex and the left posterior temporal cortex are involved in context-dependent visual size illusion, lending further support to the notion that the processing of visual size information is along the ventral visual pathway and is left lateralized.

Previous studies have indicated that the underestimation and the overestimation portions of the Ebbinghaus illusion, although very similar at behavioral level, might be supported by distinct brain mechanisms (Coren and Porac 1978; Káldy and Kovács 2003; Hadad 2018), and this point is further extended by the current study from a genetic perspective. In terms of perceptual effects, the underestimation portion and the overestimation portion of the Ebbinghaus illusion are found to be similarly influenced by genetic effects (56% and 50% variance, respectively). However, as suggested by previous studies (Kovács 2000; Káldy and Kovács 2003), visual perception of the overestimation portion, but not the underestimation portion, largely engages the intrinsic connectivity within the early visual area, and this critical difference is consolidated by the observed genetic contribution to the overestimation but not the underestimation portion in the early visual cortex.

A growing body of research has shown that genetic influences on visual cortical regions vary with stimuli and tasks. For instance, Polk et al. (2007) have shown that visual cortical responses to faces and places (i.e., houses), but not to chairs and pseudowords, are heritable in the functionally-defined ventral visual cortex. Moreover, a right-lateralized network comprising the lateral occipitotemporal and the medial parietal areas of human newborns shows stronger response to upright face-like stimuli than to inverted face-like controls (Buiatti et al. 2019). The current study demonstrated that the early visual processing related to the overestimation portion of the Ebbinghaus illusion exhibits moderate heritability. More importantly, although the feedforward and the feedback functional connectivities between the visual cortex and the temporal cortex are both negatively correlated with the perceived illusory strength, only the feedforward functional connectivity is found to be heritable. These findings, together with previous studies, support the notion that both the functional and anatomical properties of the human visual cortex are to some extent innate in nature. Meanwhile, environmental factors including visual experience play a more prominent role in modulating the neural activations in the posterior temporal region as well as its feedback functional connectivity with the early visual cortex, suggesting that genetic and environmental factors work in tandem to shape the context-dependent visual size perception. Specifically, genes and environment can take effects at different processing stages, with the former making more contribution to the early and feedforward visual processing stages while the latter contributing more to the relatively late and feedback visual processing stages. These results may enlighten future studies to bridge the gap between human brain development and G × E interactions.

In summary, the current study demonstrates heritability of visual perception of the Ebbinghaus illusion and the underlying neural mechanism using behavioral genetic methodology in combination with multichannel fNIRS. These findings provide compelling evidence that the neural computations underlying human visual size perception are susceptible to the mutual influences from genes and environment at different processing stages, and shed new light on the links among genes, environment, brain, and subjective experience. In a broad sense, our visual consciousness is shaped by an intricate interaction between genes and environment during brain development.

## Supplementary Material

SupplementaryMaterial_bhab262
