# Supplementary material for: Distinct Contributions of Genes and Environment to Visual Size Illusion and the Underlying Neural Mechanism
Source: Cereb Cortex. 2021 Aug 11;32(5):1014–23. doi: 10.1093/cercor/bhab262 (PMC8889949; doi:10.1093/cercor/bhab262)
Supplement: SupplementaryMaterial_bhab262 [file supplementarymaterial_bhab262.docx]

**Supplementary Material**

**Materials and Methods**

**Participants**

An independent group of 27 non-twin participants (13 male) with a mean age of 23.4 years (between 20 and 30 years) took part in the control experiment. All had normal or corrected-to-normal vision and gave written, informed consent in accordance with procedures and protocols approved by the institutional review board of the IPCAS, and the study adhered to the tenets of the Declaration of Helsinki.

**Stimuli and Procedure**

Five types of test stimuli including a target surrounded by four large or small inducers (T+L or T+S) as in the main experiment, four large or small inducers without a target (L only or S only), and a target only (T only) were adopted in the control experiment (Supplementary Figure 1). The stimulus parameters, the experimental procedure as well as the fNIRS data acquisition and processing were identical to those in the main experiment. There was a total of 135 trials with 27 repetitions for each condition.


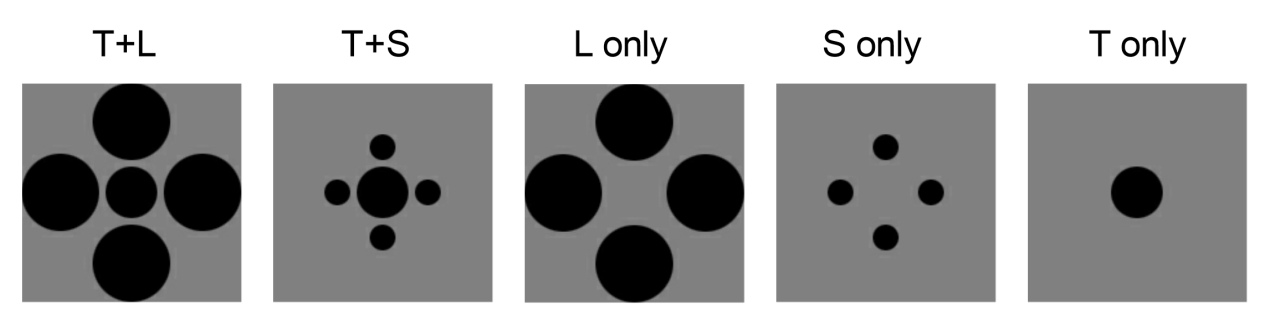


**Supplementary Figure 1.** Illustration of the stimuli adopted in the control experiment. T+L: a target surrounded by four large inducers; T+S: a target surrounded by four small inducers; L only: four large inducers without a target; S only: four small inducers without a target; T only: a target only.

**Results**

Consistent with the results of the main experiment, brain regions in the early visual cortex (ROI 1: channels #4 and #9, *t*(26) = 2.53, *P* = 0.018, *d* = 0.49) and in the posterior temporal cortex (ROI 2: channels #23 and #26, *t*(26) = 4.16, *P* < 0.001, *d* = 0.80) showed significantly greater activity to the illusory configuration of a target surrounded by small inducers relative to that of a target surrounded by large inducers [(T+S) – (T+L)]. Critically, when the oxy-Hb responses to the inducers only (S only or L only) were respectively subtracted from those to the illusory configurations (T+S or T+L), the two ROIs still exhibited greater activity to [(T+S) - S] relative to [(T+L) - L] (ROI 1: *t*(26) = 2.26, *P* = 0.033, *d* = 0.43; ROI 2: *t*(26) = 3.38, *P* = 0.002, *d* = 0.65), suggesting that the target [(T+S) - S] with large perceived size (i.e., surrounded by small inducers) compared with the identical target [(T+L) - L] with small perceived size (i.e., surrounded by large inducers) elicited significantly stronger oxy-Hb responses. This pattern of results lends strong support to the notion that the activity in the two ROIs were indeed sensitive to the perceived illusion effect because the comparison [(T+S) - S vs. (T+L) - L] eliminated any physical difference between the two test configurations and left only the perceptual illusion effect. Taken together, these results provide evidence that the differential activity in response to the targets surrounded by small and large inducers was mainly attributed to the perceptual illusion effect rather than the physical difference of the surrounding inducers.
